# Semantic text mining in early drug discovery for type 2 diabetes

**DOI:** 10.1371/journal.pone.0233956

**Published:** 2020-06-15

**Authors:** Lena K. Hansson, Rasmus Borup Hansen, Sune Pletscher-Frankild, Rudolfs Berzins, Daniel Hvidberg Hansen, Dennis Madsen, Sten B. Christensen, Malene Revsbech Christiansen, Ulrika Boulund, Xenia Asbæk Wolf, Sonny Kim Kjærulff, Martijn van de Bunt, Søren Tulin, Thomas Skøt Jensen, Rasmus Wernersson, Jan Nygaard Jensen

**Affiliations:** 1 Novo Nordisk Research Centre Oxford, Novo Nordisk Ltd., Oxford, United Kingdom; 2 Intomics A/S, Kgs. Lyngby, Denmark; 3 Novo Nordisk A/S, Bagsværd, Denmark; 4 DTU Health Tech, Technical University of Denmark, Kgs. Lyngby, Denmark; Clemson University, UNITED STATES

## Abstract

**Background:**

Surveying the scientific literature is an important part of early drug discovery; and with the ever-increasing amount of biomedical publications it is imperative to focus on the most interesting articles. Here we present a project that highlights new understanding (e.g. recently discovered modes of action) and identifies potential drug targets, via a novel, data-driven text mining approach to score type 2 diabetes (T2D) relevance. We focused on monitoring trends and jumps in T2D relevance to help us be timely informed of important breakthroughs.

**Methods:**

We extracted over 7 million *n*-grams from PubMed abstracts and then clustered around 240,000 linked to T2D into almost 50,000 T2D relevant ‘semantic concepts’. To score papers, we weighted the concepts based on co-mentioning with core T2D proteins. A protein’s T2D relevance was determined by combining the scores of the papers mentioning it in the five preceding years. Each week all proteins were ranked according to their T2D relevance. Furthermore, the historical distribution of changes in rank from one week to the next was used to calculate the significance of a change in rank by T2D relevance for each protein.

**Results:**

We show that T2D relevant papers, even those not mentioning T2D explicitly, were prioritised by relevant semantic concepts. Well known T2D proteins were therefore enriched among the top scoring proteins. Our ‘high jumpers’ identified important past developments in the apprehension of how certain key proteins relate to T2D, indicating that our method will make us aware of future breakthroughs. In summary, this project facilitated keeping up with current T2D research by repeatedly providing short lists of potential novel targets into our early drug discovery pipeline.

## Introduction

Drug discovery often involves prioritising potential drug target candidates based on a combination of researchers’ knowledge, data from wet-lab experiments, and *in-silico* predictions. The researchers’ knowledge typically comes from their own work as well as publicly available scientific literature. Due to recent technological advancements, publicly available knowledge bases are growing exponentially via large-scale omics, human genetics studies, imaging projects, and electronic health records [1, 2]. Thus, for the most well-studied diseases thousands of relevant articles are available. Text mining is one of the most common tools to help choosing which articles to read, and extract explicit facts, but also to combine independent pieces of knowledge, thereby prioritising resources in the early drug discovery phase.

Type 2 diabetes (T2D) is a chronic disease with about half a billion diagnosed patients, and probably the seventh leading cause of death due to its many co-morbidities (such as blindness, kidney failure, heart attacks, stroke, and lower limb amputation) [3]. T2D is a multi-factorial disease associated with many proteins (from 30 in OMIM [4], through more than 200 genetic loci [5], to over 3,000 postulated associations in OpenTargets [6]). The continuous growth of our understanding of signalling events in T2D, and therefore relevant literature, is considerable. No single person, group, or even large organisation is able to keep up with all details, but is instead more likely to catch the high impact literature only. In 2018 more than 1 million articles were indexed by PubMed, or around 20,000 articles per week, of which we estimate 1–2%, equivalent to hundreds per week, to be relevant for T2D.

A simple keyword search for T2D synonyms will of course find all known articles where the association to T2D has already been established, but may also find articles on, for example, patient care that are less interesting in a drug discovery context. Such articles will dilute the overall results and the ability of text mining to support drug target identification. On the other hand, many relevant papers regarding the complex underlying biology may not mention T2D synonyms, either because the association is not yet established, or it may be understood from the context. Such papers may investigate co-morbidities of T2D or related metabolic processes, and may therefore not be found in a keyword search.

Text mining could be described as the process of discovering, and capturing, knowledge from a large number of unstructured texts, and it is being applied to more and more problems in drug discovery [7]. Text mining tools vary from simple over-representation statistics between two sets of keywords like [8] to more complex NLP approaches like [9]. To date, most text mining tools in the biomedical field are specialised to specific tasks [10–13]. Some systems, e.g. BioReader [14], find both direct and indirect associations by considering large numbers of search terms using machine learning algorithms, and a recent application [15] applied the Word2vec [16] algorithm to materials science literature successfully. Other tools are designed to work on a general level, such as IBM Watson [17] and SciBite’s TERMite [18].

In order to find associations you have to specify what you are looking for, i.e. your vocabulary, in a Named Entity Recognition (NER) step. This means that you are inherently limited to those concepts in the specified vocabulary. There has been focus on solving this out-of-vocabulary problem for example by using machine learning to determine the vocabulary from data using *n*-grams [19]. Alternatively, ToPMine [20] (and tools built on this like Moliere [21]) is a purely data-driven approach which focus on identifying significant, frequent phrases.

Another approach is the ‘question/answer’ approach in which you focus on predicting the next word(s) given a certain set of preceding words [22].

There are also methods trying to find the implicit link between two concepts based on the ABC co-occurrence model, where if A and B are co-mentioned, and B and C are co-mentioned, you assume there is a link between A and C, for example [23] or similar approaches like [24]. Relying on the MeSH annotation [25] for individual papers instead of text mining, [26] studies co-annotations, aiming at forming new medical hypotheses based on evolutional trajectories of their embeddings for different five years’ time slices.

In the present work we focus on, potentially right after publication, linking proteins and relevant mechanisms learned from the literature itself to diseases, in this case T2D, as proteins are the actual agents driving the underpinning physiological mechanisms; this is the very heart of molecular medicine. Several projects seek to associate genes/proteins to diseases; two popular, such databases, OpenTarget and DisGeNET [27] currently list thousands of T2D associated proteins. More specifically, DisGeNET currently lists 1,671 genes when querying for T2D, while Open Targets currently lists 3,346 associated genes. These numbers are growing as more knowledge is disseminated. In addition, some disease proteins yet remain to be well described in literature (see S1 File S1.1). This makes it a challenge to determine appropriate statistical criteria for disease associations. However, only a handful of proteins are currently used as targets for drugs for T2D, so we reason there is still a potential for identifying novel drug targets.

**Fig 1:**
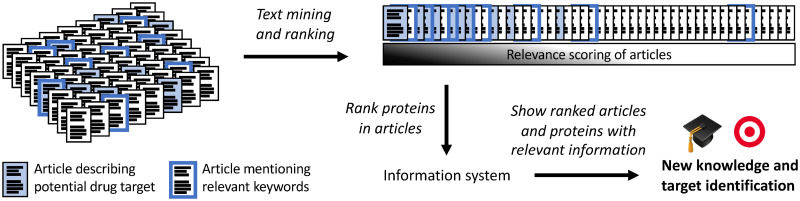
Using text mining to guide early drug discovery. Keyword searches for publications, like in PubMed, may find thousands of articles (borders highlighted), including those less relevant to drug discovery, e.g. on the care of patients. Yet, articles describing potential drug targets (background coloured) risk not being found as the disease association may be implicit or not yet established. To address these issues, articles are ranked using text mining, focusing on molecular biology without requiring direct mentioning of the disease. Proteins are then ranked, and the information system shows the articles with relevant terms highlighted in addition to pertinent information, providing a convenient workflow for advancing knowledge and identifying drug targets.

Here we present a project that addressed the problem of quantifying, and at the same time providing an explanation for, the relevance of papers for T2D. This work is being used to guide the discovery of novel T2D drug targets. Fig 1 illustrates, on a high level, how we used our text mining scores of papers’ T2D relevance to help obtain new knowledge and identify new targets. Similar to other projects, it prioritised among the thousands of proteins already tentatively linked to T2D, glucose control, and/or insulin resistance. A benefit to our approach is that we broadened the definition of the disease by learning and focusing on the underlying biological mechanisms. The broadening was done by basing our relevance score on a purely data-driven detection of multi-word terms, *n*-grams, and their clustering into what we call ‘semantic concepts’, rather than a set vocabulary. The molecular focus and T2D relevance was ensured by weighing the concepts according to their enrichment with articles mentioning one, or more, of the 100 proteins most significantly associated to T2D via simple synonym searches. We could then identify T2D relevant proteins, potentially even before they have been explicitly co-mentioned with T2D. Each protein was scored based on five years’ worth of articles. It is well known that certain proteins have, at some points in time, started receiving more attention in the T2D relevant literature. To face the unsolved challenge of ensuring awareness of such proteins, we investigated trends and changes in T2D relevance. We introduced the term ‘high-jumpers’ for proteins that significantly change their rank in comparison to the distribution of rank changes seen over the previous years. Utilising existing infrastructure, the project continues to present interesting articles and proteins on a weekly basis, as it is essential for early drug discovery to be made timely aware of potential novel drug targets.

## Methods

We text mined PubMed abstracts using NER to find the proteins mentioned in each abstract. For our project we used a custom text mining engine implemented in C and Python (see S1 File S1.1), but any NER system that can find proteins mentioned in a text using a set of well-curated synonyms (e.g. PubTator [10–12], TERMite [18], or the system used at JensenLab [13]) can be used by the method we present. PubMed abstracts were also mined for mentionings of T2D, and we used Fisher’s exact test to measure how co-mentioned a protein was with T2D (see S1 File S1.2 for details). All proteins were ranked by their *p*-values for being related to T2D (see S1 File S1.3 for details), and we chose to denote the 100 most significantly associated as ‘core proteins’ as they have well-established and well-known associations to T2D (according to the literature captured in PubMed).

We used these 100 core proteins to characterise our biological area of interest and proceeded to construct an article scoring scheme by favouring terms co-mentioned with the 100 core proteins, and used these individual article scores to prioritise proteins according to T2D relevance.

A rigorous mathematical description of our methods and statistical analyses is found in S1 File sections S1.1 through S1.10.

### Scoring T2D relevance of articles using semantic concepts

**Fig 2:**
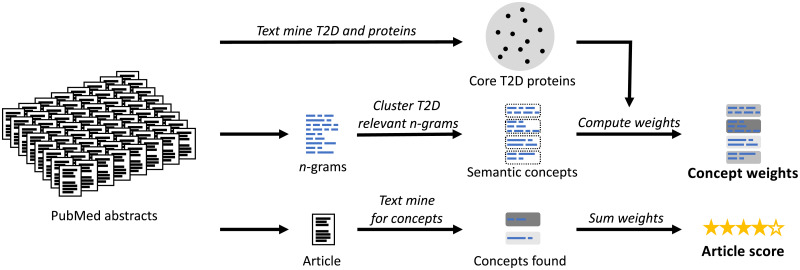
Scoring T2D relevance of articles. Named entity recognition is used to find the 100 core T2D proteins that are most significantly co-mentioned with T2D in PubMed abstracts. Commonly used word sequences (*n*-grams) are learned from the abstracts and T2D relevant ones are clustered into ‘semantic concepts’, if they are textually similar, or used in the same context. The semantic concepts are assigned weights based on how often they are co-mentioned with the core T2D proteins. All articles, old and new, are then scored by summing the weights of the semantic concepts found in them.

Fig 2 outlines our data-driven method for scoring article abstracts for T2D relevance. We focused on the relevant biology by revolving the analysis around the 100 core T2D proteins, and we broadened the search by looking for phrases related to the core T2D proteins rather than the disease directly. As will be further discussed under ‘T2D relevance of articles’ in the ‘Results’ section, we could in this way give high scores to articles with abstracts that did not mention T2D explicitly, but instead used many of the same phrases and terms as abstracts mentioning proteins known to be related to T2D. In addition to expanding the scope of the biological space, e.g. our understanding of how the disease works, this meant that we could potentially identify proteins before they become well known in a T2D context.

To broaden our search beyond articles mentioning T2D explicitly, and to focus on articles on molecular biology (in contrast to e.g. care of diabetes patients), we set out to find articles that discuss the same biology in their abstracts as articles mentioning the 100 core T2D proteins. For this, we needed a set of entities and synonyms that could be used to properly characterise the abstracts. Even though a large effort has been put into creating ontologies like MeSH and UMLS [28] for biomedical terms as well as repositories like Semantic MEDLINE database [29], these resources did not encompass all relevant phrases. Instead, we decided to extract a terminology directly from PubMed in a purely data-driven manner where we identified frequently used sequences of words, *n*-grams (see S1 File S1.4).

Many *n*-grams, even for different sizes of *n*, refer to the same semantic object (e.g. ‘lower glucose’ and ‘lowers blood sugar level’). Considering all these *n*-grams individually diminishes the overall statistical power. We therefore grouped similar, and related, *n*-grams into what we coined ‘semantic concepts’. We first constructed a similarity measure to join textually similar *n*-grams that are spelled slightly differently, or have their words rearranged, e.g. ‘type 2 diabetes’ and ‘diabetes type II’ (see S1 File S1.5 for details). Secondly, we measured the semantic relatedness between *n*-grams by computing how often they co-occur in relevant articles by constructing a similarity matrix where each cell contains the over-representation ratio for the two *n*-grams corresponding to the row and the column (see S1 File S1.6). The rationale for using this similarity matrix, was that if, say, two rows in the matrix were very similar, the two *n*-grams would be co-mentioned with the same set of *n*-grams and hence tend to be used in the same context, and would be likely to share the same ‘meaning’, even though they need not be synonyms (for example ‘leptin treatment’ and ‘elevated leptin’ are not synonyms but are considered part of the same semantic concept). We finally used the MCL algorithm [30] to group *n*-grams into semantic concepts based on these two similarity measures simultaneously (see S1 File S1.7 for details).

Our semantic concepts are therefore clusters of *n*-grams that have both some degree of string similarity as well as similar co-mentioning with other *n*-grams; i.e. they behave the same way semantically and look alike string-wise. It is important to note that the individual *n*-grams for each semantic concept need not be synonyms, but they are expected to be equally relevant in a given context (e.g. ‘increased glucose’, ‘decreased glucose’ and ‘total glucose’ are clustered together by our method along with other glucose related *n*-grams).

We assigned weights to the semantic concepts by first computing how much more than expected by random they were co-mentioned with the core set of 100 T2D proteins. For each of the core proteins, and for each semantic concept, this ratio was log-transformed to get the what is known as the pointwise mutual information [31], and the weight for each semantic concept was computed as the average across all the core proteins (see S1 File S1.8 for details). An article was scored by simply taking the sum of the individual weights of all the semantic concepts with *n*-grams in the article, thereby broadening our search beyond articles explicitly mentioning T2D, and at the same time zooming in on articles related to the molecular biology of T2D. In other words, an article will get a high score, if it uses similar words as articles mentioning the 100 core T2D proteins, and the more specific these words are, the higher the score.

### Determining T2D relevance of proteins

**Fig 3:**
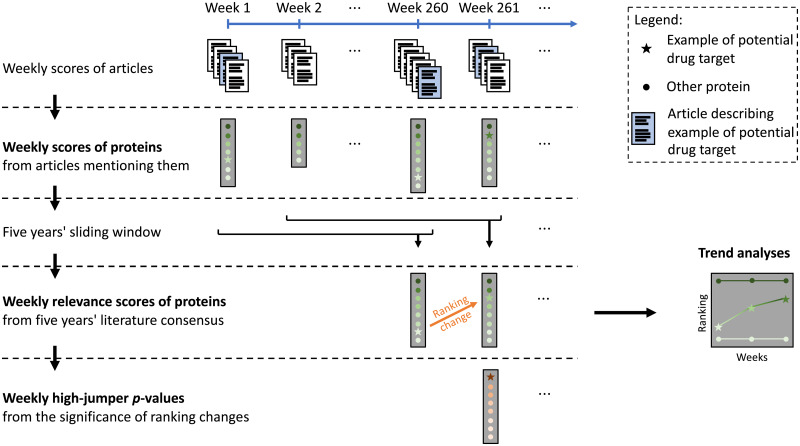
Scoring T2D relevance of proteins. Articles from PubMed are grouped by the week they were added to the database. The T2D relevance of proteins is prioritised in different ways: 1) the highest T2D relevance score for an article mentioning the protein and published a given week; 2) these scores for the last five years combined into one consensus score (updated weekly using a sliding window); and 3) *p*-values assessing a protein’s change in the overall ranking, pinpointing high-jumping proteins that are suddenly mentioned in a T2D relevant context. In addition, looking at long-term trends of the combined scores may reveal interesting biology or proteins.

In this project we computed two different T2D relevance scores for proteins, both based on the scores of the articles mentioning them (see Fig 3).

#### Five years’ T2D relevance score

As the weekly score of a protein we used the highest score of an article mentioning the protein in that particular week. We then ranked the previous 260 weekly protein scores in order to determine which proteins are the most relevant to T2D as per the combined recent research (in a five years’ sliding window). The 40 highest were then weighted so the highest contributed most to the T2D relevance score for the protein. In this way, contributions had to be spread out over time and not all come from the same week (e.g. a single conference’s proceedings). This is described more formally in S1 File S1.9. By shifting the time period one week at a time, we could retrospectively visualise trends in T2D relevance for individual proteins.

#### Weekly high-jumping proteins

In order to find novel T2D proteins, we focused on the current week and identified proteins with high upward jumps and the abstracts causing these. This expanded our more traditional weekly literature survey, by selecting a different set of articles, and by focusing on the most important proteins in these (for example insulin tend to be mentioned in all articles but is not interesting from a new drug target point of view).

Seeing a top ranking protein improve its rank by, say, three places is not very likely, and is certainly something we should be made aware of if it happens. It is more likely, but less interesting, for a protein further down the list to improve its rank by three places. A protein that jumps hundreds or thousands of places may be relevant, depending on how typical such jumps are.

To address this, the empirical distribution of jumps for each initial rank was used, i.e. we looked back in time and observed both the jumps and their starting ranks. When a protein’s weekly rank changed, we consulted the distribution corresponding to its initial rank to get a *p*-value for the jump. However as not all jumps were observed, we smoothed the distributions by applying a sliding window (see S1 File S1.10 for details). Hence, a jump in a proteins rank got a low *p*-value if the protein was unexpectedly mentioned in an article with high T2D relevance score, based on the combined previous knowledge about the protein.

### Supporting early drug discovery

On a weekly basis, all new articles abstracts from PubMed were text mined and ranked by our scoring scheme that prioritised abstracts with the same semantic concepts as those mentioning the core T2D proteins. The high ranking article abstracts were presented in the web interface where protein synonyms, diseases, drugs, and anatomical entities were highlighted in order to easier derive novel biological insight.

The same framework was also used to display the most relevant proteins for a given week, based on the protein scores described previously. In order to be kept informed of new trends in T2D research we ranked the proteins according to their weekly scores. In addition, in order to find novel targets before they are T2D associated, we also highlighted the ‘high-jumping’ proteins, i.e. the proteins that were suddenly ranked more T2D relevant than previously (as described in the previous section). For each featured protein we both retrieved annotations and linked to other internal information systems with pertinent details, such as if this protein is already known in a T2D context or not.

## Results

Of the 30.3 million articles registered in PubMed in November 2019, we text mined the 19.8 million (65%) with both a title and an abstract. From these abstracts we first identified and reviewed the set of the 100 most T2D associated proteins (see S1 File S1.3) that was the starting point for our main results described in the following sections.

### T2D relevance of articles

We broadened the scope of the disease biology, trying to better characterise T2D by implementing an unsupervised method of generating, and evaluating semantic concepts with thousands of these contributing towards the article scores. Among the more than 7 million *n*-grams we extracted (see S1 File S1.4), 235,382 were over-represented with T2D and these were clustered into 48,381 semantic concepts.

The use of unsupervised detection of semantic concepts directly from PubMed rather than using traditional vocabularies enabled us to capture terms that we might not necessarily have included otherwise. Investigation of the important concepts expanded our understanding of the disease, the mechanisms behind it, and the relevant tissues.

For each semantic concept we computed a weight that was used to score individual articles (see the section ‘Scoring T2D relevance of articles using semantic concepts’ in ‘Methods’ and S1 File S1.8). Table 1 shows the top 50 semantic concepts we found (and their assigned weights), each represented by their most common *n*-gram. They all had weights close to 1, meaning that they were on average co-mentioned 10 times as often as expected by random with one of the top 100 core proteins. The highest scoring semantic concept is a cluster of *n*-grams about glucose metabolism. The most frequently used *n*-gram in this concept is simply: ‘glucose homeostatis’ but since the literature has a natural focus on describing and understanding disease-states, the other *n*-grams reference dysfunction of this metabolic process, hence the concept also includes ‘disturb glucose homeostasis’, ‘disturbed glucose homeostasis’, ‘defective glucose metabolism’, ‘impair glucose homeostasis’, and ‘impairs glucose homeostasis’.

**Table 1:** Top 50 semantic concepts for T2D. The semantic concepts with the highest weights for T2D are focused on glucose homeostasis or tolerance as well as insulin resistance, sensitivity, levels, secretion, and the insulin receptor substrate 1 before the db/db mouse model and T2D itself.

Rank	Most common *n*-gram	Weight	Abstracts	Rank	Most common *n*-gram	Weight	Abstracts
1	glucose homeostasis	1.208	8,916	26	fasting insulin	0.975	6,865
2	insulin resistance	1.155	59,462	27	leptin levels	0.962	20,700
3	insulin sensitivity	1.132	21,599	28	insulin glucose	0.961	2,550
4	aims hypothesis	1.130	5,728	29	plasma glucose concentration	0.961	5,093
5	glucose tolerance	1.121	16,714	30	diet induced obese	0.960	1,287
6	insulin receptor substrate 1	1.113	2,939	31	obese diabetic mice	0.960	611
7	insulin secretion	1.104	17,655	32	phase insulin secretion	0.959	1,743
8	improved glucose tolerance	1.099	2,330	33	improve insulin resistance	0.958	1,034
9	insulin levels	1.093	10,194	34	glucose tolerance tests	0.957	2,366
10	db db mice	1.092	4,104	35	cell counting kit	0.957	16,141
11	type 2 diabetes mellitus t2dm	1.088	13,215	36	oral glucose challenge	0.955	1,583
12	increased insulin sensitivity	1.074	2,475	37	diet induced	0.946	7,837
13	insulin resistant	1.071	5,928	38	obese mice	0.943	5,735
14	type 2 diabetes	1.070	91,290	39	diet induced changes	0.940	1,895
15	glucose metabolism	1.046	26,156	40	increased insulin	0.938	1,098
16	impaired glucose tolerance	1.041	20,710	41	plasma insulin levels	0.937	5,026
17	high fat diet	1.038	16,674	42	glucose stimulated insulin secretion	0.937	3,029
18	β cell function	1.027	2,591	43	insulin resistance ir	0.935	5,129
19	diabetes t2d	1.022	12,100	44	insulin receptor substrate	0.934	1,589
20	plasma glucose	1.017	13,480	45	insulin glucagon	0.931	5,633
21	induced insulin resistance	1.005	2,432	46	insulin tolerance test	0.930	1,393
22	beta cells	0.997	20,780	47	insulin receptor	0.929	9,195
23	high fat diet hfd	0.993	9,226	48	homeostasis model assessment	0.929	6,774
24	ob ob mice	0.987	4,394	49	lipid metabolism	0.928	31,435
25	plasma insulin	0.984	8,133	50	insulin resistance homa ir	0.927	5,059

We manually inspected the 1,000 highest scoring article abstracts (since 2014) to assess their relevance for T2D. Of these, 406 mentioned T2D explicitly, 181 had the MeSH term ‘Diabetes Mellitus, Type 2’ (with 168 overlapping). The remaining 581 abstracts were often about insulin resistance, or a well-known co-morbidity such as obesity, non-alcoholic fatty liver disease, diabetic neuropathy, kidney, or cardiovascular disease. Another prevalent theme among the inspected abstracts is that they talk about genes (variation, expression or knockout) or experiments in animals. Occasionally, gestational diabetes, type 1 diabetes, or streptozotocin induced diabetes would instead be the focus on the abstract.

We also inspected abstracts with the ‘Diabetes Mellitus, Type 2’ MeSH term, but with low scores, and found that these articles were often about case reports, the care of T2D patients, or it was not evident from the title or abstract alone why the article was tagged with the MeSH term for T2D, for example [32] which discusses an extension of a patient questionnaire that was then evaluated on T2D patients.

As can be seen in Table 1 the system separated semantic concepts of a similar nature like ‘type 2 diabetes’, ‘type 2 diabetes mellitus t2dm’, and ‘diabetes t2d’; even though these would normally be considered synonyms for the same biological concept. While this may at first seem surprising, we do note that these concepts get similar, high weights. In general, the data-driven approach generated higher resolution concepts compared to manually maintained ontologies, enabling us to separate terms and thereby gave us a greater understanding of the biological space. For example, ‘glucose tolerance’ had the 5th highest weight, ‘improved glucose tolerance’ had the 8th highest, while ‘normal glucose tolerance’ had the 54th highest weight (not shown).

As a curiosity we noted that the semantic concept with the 4th highest weight contained the *n*-gram ‘aims hypothesis’ because abstracts from the journal Diabetologia all have the heading ‘aims/hypothesis’. Also, the 35th highest ranking semantic concept is represented by the *n*-gram ‘cell counting kit’ in the table as this is the most prevalent *n*-gram for the semantic concept that also encompasses more than 100 different *n*-grams including ‘pancreatic β cells’. Apart from these two cases, all the semantic concepts with the highest weights are, as expected, highly relevant for T2D (or obesity).

### T2D relevance of proteins

**Fig 4:**
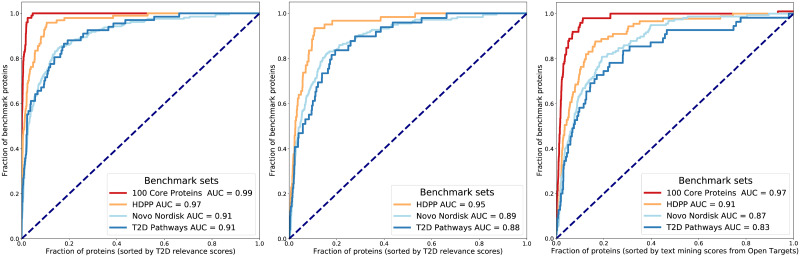
Benchmarking the T2D protein relevance scores. *Left:* The ROC curves show that we find a) the 100 core proteins used to characterise our area of interest (with an AUC very close to 1), b) the 100 diabetes relevant proteins identified by the HDPP project (3 of these proteins are not be found by text mining), c) a set of 209 proteins of particular interest to the project, and d) 66 proteins from public type 2 diabetes pathways (KEGG and WikiPathways) or annotated in UniProtKB as relevant for T2D. *Centre:* Here, the 100 core T2D proteins are removed from each benchmark set (62 remain in HDPP, 181 in the internal set, and 47 in the T2D pathways), which obviously lead to slight AUC reductions. *Right:* The curves when using text mining scores from Open Targets [39].

As we computed T2D relevance scores for proteins from the last five years’ of literature, we expected well-known T2D proteins to get the highest scores. We benchmarked this by examining how well the scoring scheme for this project was able to find three sets of T2D proteins via ROC curves; 1) T2D proteins from the Human Diabetes Proteome Project [33], 2) a manual benchmark list of proteins selected for this project, and 3) proteins from public T2D pathways (KEGG [34–36], WikiPathways [37]) or annotated in UniProt [38] as relevant for T2D (Fig 4, left and centre). All had AUCs above 0.88, i.e. using our scoring scheme, the set of high scoring proteins was clearly enriched for the well known T2D proteins. We also compared our scoring to the text mining scores from Open Targets [39] (Fig 4, right) and noted that we have sligthly higher AUCs.

Table 2 shows the 100 proteins with the highest relevance scores. There is, not surprisingly, a large overlap with the 100 core proteins. The proteins, and papers, that we had not previously flagged were investigated, and almost all of them were deemed interesting. This shows that this automatic data-driven approach works, as it keeps the focus on T2D while at the same time expanding the bio-molecular knowledge space around T2D.

**Table 2:** Top 100 most T2D relevant proteins from the last 5 years according to our scoring scheme, sorted alphabetically by gene name. The 54 proteins with an asterisk (*) are also part of the set of 100 core T2D proteins.

ACOX1	BCL2	DECR1	GCGR*	HSD11B1*	ITGAX	NAMPT*	PNPLA2	RBP4*	SOCS3
ADIPOQ*	CASP3	DGAT1	GCK*	HSPA5	LDLR	NFE2L2	POMC	REN*	SREBF1*
ADIPOR1*	CAT	DPP4*	GHRL*	ICAM1	LEP*	NFKBIA	PPARA*	RETN*	SREBF2
AGT*	CCK	EEF1A2*	GIPR*	IGF1*	LEPR*	NOS2	PPARD*	SCD	STAT3
AKT1*	CCL2*	FABP4*	GLP1R*	IL10	LPL*	NOS3*	PPARG*	SERPINE1*	STK11
AKT2	CD36*	FASN	GOT2	IL1B	MAPK3	NOX4	PPARGC1A*	SIRT1	TGFB1
APOA1*	CD68	FGF21*	GPX1	IL6*	MAPK8	NR1H3	PRKCE	SLC2A1*	TLR4
APOB*	CEBPA	FOXO1*	GSK3B*	INS*	MGAM*	PCK1	PTEN	SLC2A2*	TNF*
APOC3*	CPT2	G6PC*	HMGCR	INSR*	MLXIPL	PDK4	PTGS2	SLC2A4*	UCP1*
APOE*	CRP*	GCG*	HMOX1	IRS1*	MTOR	PDX1*	PTPN1*	SLC5A2*	VCAM1*

#### Trends in T2D relevance

**Fig 5:**
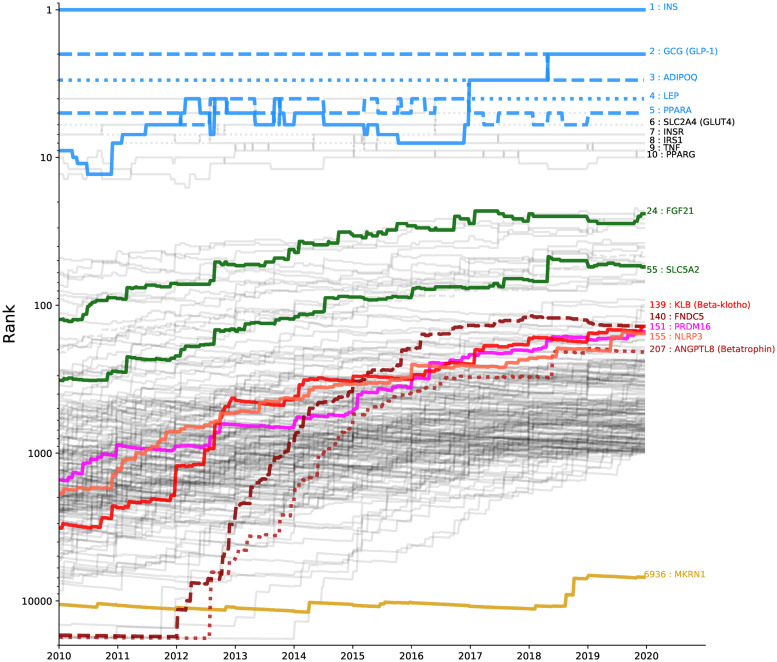
Evolution and jumps of protein rankings. For a selection of proteins the curves show the ranks during the last 10 years. We include the top 10 proteins at the time of our analysis, where the top 5 are coloured in blue. Additionally, 180 proteins that improved their ranks with a factor of at least 2 are included. The green curves show FGF21 and SLC5A2, the 2 proteins among the top 100 which improved their ranks 5 times, or more. The redish curves show proteins improving ranks at least 10 times, ending up among the top 300 proteins: KLB (beta-klotho), FNDC5 (dashed), PRDM16, NLRP3 and ANGPTL8 (betatrophin; dotted). In addition, the golden curve near the bottom shows MKRN1, an example that has 2 significant weekly jumps.

It is known that pharmacology for T2D has advanced rapidly during the last 10 years and it is now possible to target different pathophysiological defects (as the existing classes of T2D drugs do). Accordingly, we observed that for certain proteins the strength of their association to T2D change over time; e.g. going from the initial discovery to a more detailed understanding of the protein’s biological role. To study this evolution, we plotted the protein ranks as they changed over the last 10 years. Fig 5 shows these curves for the proteins that improved their ranks two-fold, or more, over the period plus the 10 most associated proteins. We found these trend curves to be generally representative of the development in T2D research, as we will show in the following sections by highlighting a few examples.

As expected, insulin is constantly the top ranking T2D protein (it’s effective in all disease stages and necessary to achieve glycemic control [40]). The second place belonged to adiponectin for more than 8 years. This protein is secreted by adipose tissue and is a metabolic regulator of glucose control and breakdown of fatty acids [41]. In addition, adiponectin can reverse insulin resistance in mice in combination with leptin [42]. Adiponectin is therefore strongly involved in T2D processes. In the beginning of 2018 its second place was taken by glucagon (GLP-1).

The high ranking of leptin can be explained by the fact that it was discovered as the hormone regulating appetite in obese mice, which is a known co-morbidity to T2D, but also raised considerable interests within the T2D research community for its possible implication in sustaining insulin resistance [43] and ability to positively influence plasma glucose levels in diabetic mice [44]. Leptin was a clear third from the beginning of our trend analysis until the end of 2016 where glucagon (GLP-1) caught up.

The 5th place is PPARA (Peroxisome proliferator-activated receptor alpha) which since 2000 has been known to improve insulin sensitivity [45]. Eleven genetic variances (11 SNPs) were associated with response to an anti-diabetic drug (acarbose) in 2007 [46]. The recent development around PPARA is in large part due to [47], as well as [48] and [49].

On a global level we saw considerable more movement than we expected within the 100 most associated T2D proteins. To illustrate this, we overlaid the 2 proteins that improved their rankings at least 5 times and ended up among the top 100 (green curves, Fig 5). For example, FGF21 (fibroblast growth factor 21) is implicated in uptake of glucose in adipose tissue, but not in other tissues [50], it had an initial rank of 125 at the beginning of 2010, yet managed to improve its rank 5.2 times ending up at rank 24 by the end of 2019. Another significant mover that came from well outside top 100 (place 322) but settled within top 100, at place 55, was SLC5A2, or sodium/glucose cotransporter 2, responsible for retention of blood glucose by re-absorption of glucose in the kidneys [51].

#### Detecting jumps in T2D relevance

Although many likely T2D targets are part of the top scoring proteins, they do not represent a source of novel disease associations. Thus, we were also interested in suggesting articles that either establish a ‘first’ strong connection to the disease or greatly improve an already existing weak association. We expected traditional text mining approaches based solely on numbers of co-mentionings to only find well-studied proteins with obvious disease associations, as novel proteins would not have sufficiently many mentionings to be found. Thus, to allow novel proteins and valuable research findings to be uncovered, even with few mentionings, we investigated if a protein was suddenly mentioned in a T2D relevant context.

To investigate how proteins changed their T2D rank each week, and to study when the most significant jumps occur, we computed the empirical distribution of jumps given the initial rank of a protein and then investigated the most extreme changes. In general, most proteins retained their rank from week to week, and hence the median change is very close to zero. This is the result of either no new published associations, or because there were no papers with large enough scores to improve the overall protein rank. Only a few proteins compete for a top placement and most are irrelevant for early drug discovery since they have already been examined as potential drug-targets. When proteins do change rank, the typical jump was −1 caused by a large positive jump of another protein. An interesting minority of changes were jumps for proteins jumping hundreds of places, signifying a specific publication with a large impact towards our understanding of the protein’s function in T2D context.

**Fig 6:**
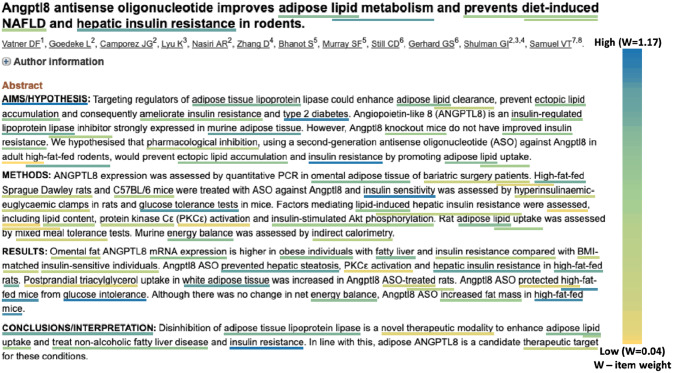
An example of a high scoring article causing a significant jump. This paper on angiopoietin-like 8 caused the rank of ANGPTL8 to jump up 107 places from rank 312 to 205, corresponding to a *p*-value of 0.000038 in week 22 of 2018. The text is reprinted from the abstract of [58], licensed under the Creative Commons Attribution 4.0 International License [59].

Incidentally, angiopoietin-like 8 (ANGPTL8), previously known as RIFL or betatrophin, was identified as an ‘insulin target’ in 2012 by [52] causing a jump of 8,645 places from rank 17,955 to 9,310, corresponding to a *p*-value of 0.000075 (the first jump for the dotted, dark red curve in Fig 5). It was considered a potential drug-target due to its alleged role as a pancreatic β cell peptide growth hormone [53]. Although this claim was quickly retracted [54] as other groups failed to replicate the finding, research still continued into ANGPTL8’s function due to its negative correlation with improved fasting glucose levels [55], [56] and its ability to regulate serum triglyceride levels [57]). This combined research led to a total placement in the top 400 in 2018 when it was discovered that disinhibition of adipose tissue lipoprotein lipase is a novel therapeutic modality of ANGPTL8, to enhance adipose lipid uptake and treat non-alcoholic fatty liver disease and insulin resistance [58]. This finding (see Fig 6) caused another significant jump in rank corresponding to the almost vertical, dashed, red line in mid 2018 in Fig 5.

**Fig 7:**
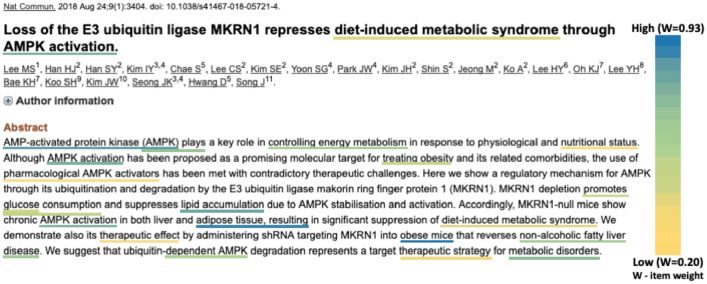
An example of a significant high-jumping article. This single article caused MKRN1 to jump from rank 10,853 to 9,082 with a *p*-value of 0.00087 in week 34 of 2018. The text is reprinted from the abstract of [60], licensed under the Creative Commons Attribution 4.0 International License [59].

Another example of an interesting high-jumping event is an article that does not mention T2D explicitly, but argues that AMPK degradation represents a therapeutic strategy for metabolic disorders. It describes an observation where the deletion of MKRN1 promotes glucose consumption and suppress lipid accumulation. The main focus of the abstract is on obesity and its co-morbidities, such as nonalcoholic steatohepatitis (NASH) [60]. The article’s score is low enough that it was only the 55th most T2D relevant paper in week 34 of 2018, so without the high-jumper analysis we could have missed it (Fig 7 depicts the abstract text, its semantic concepts, and their corresponding weights).

This article corresponds to the first (*p* = 0.00087) of the two significant, golden coloured jumps seen in the second half of 2018 Fig 5. The second jump (*p* = 0.00019) was for the 11th most T2D relevant article in week 41 which concludes with ‘*implicating MKRN1 as a possible therapeutic target for metabolic syndromes, such as obesity, type II diabetes, and fat liver diseases*’ [61]. Therefore our high-jumping analysis correctly suggested MKRN1 as a new T2D target before it was explicitly stated, and before the suggestion that glycemic regulation by AMPK could be a therapy for T2D [62].

## Discussion

By integrating this project into the pre-existing, weekly literature surveys we made it easier to investigate the literature in multiple ways, thereby accelerating and empowering the early drug discovery process for T2D.

Our data-driven, automatic scoring of papers using text mining guided researchers to papers they may not otherwise have been aware of. Automatic detecting and weighting *n*-grams prioritised papers that did not mention T2D explicitly, but rather focused on the molecular biology, as we built our scoring scheme around the 100 most significantly disease associated proteins. In particular, we found and used *n*-grams that were not present in relevant dictionaries. A possible limitation of this part of our method is that the clustering of *n*-grams needs to be done for each disease individually, as only *n*-grams co-mentioned with T2D were considered. We believe however, that we can further enhance our method and get a set of universal semantic concepts. In addition, the grouping of *n*-grams into semantic concepts occasionally led to some artefacts, such as splitting synonyms across multiple concepts, or grouping *n*-grams that were not equivalent. Nonetheless, we believe we obtained a more fine-grained understanding of the underlying biological processes from investigating the semantic concepts separating for instance the positive, negative, and neutral regulation of a specific function. A benefit of our scoring scheme was that it is easily explainable as it sums the concepts’ weights, each of which can be visualised as per e.g. Fig 7.

The article scoring scheme we introduced here aims at giving high scores to papers leading up to establishing new associations between a protein and T2D biology. These individual article scores were turned into weekly protein scores, that were investigated across a five years’ window, and we have shown that this integrated score could identify the well-known T2D proteins with a slightly better performance than with the text mining scores from Open Targets. By focusing on different sliding windows we found well-known past changes of direction in T2D research. While we have thoroughly vetted the NER for the 100 T2D associated proteins our scoring scheme is based on, the possibility of false positive NER results represents a limitation to our method when identifying proteins. We faced this by incorporating NER results and markup in our framework for displaying the outcome of our analysis, making false positives easier to discern.

Our relevance scoring of proteins, and subsequent identification of high-jumping proteins, overcomes the inherent issue in protein-centric text mining that the best characterised proteins typically dominate the results. It is a major strength of our method that we can detect T2D relevance in single papers, even for proteins with no strong, prior association to T2D. These novel proteins are more intriguing from a target identification point of view, but at the same time require more validations. Typically, we investigated a handful of high-jumping proteins each week. As we re-discovered previous case-stories we believe we have a useful surveillance mechanism in place likely to make us aware of scientific breakthroughs early on.

The fact that we were encouraged to extend the project to cover other disease areas is perhaps the best indication that it contributes positively to the early drug discovery process.

## Supporting information

S1 File (PDF)
