# Supplementary material for: Semantic text mining in early drug discovery for type 2 diabetes
Source: PLoS One. 2020 Jun 15;15(6):e0233956. doi: 10.1371/journal.pone.0233956 (PMC7295186; doi:10.1371/journal.pone.0233956)
Supplement: S1 File — (PDF) [file pone.0233956.s001.pdf]

## S1.1 Named entity recognition

Synonyms for genes and proteins were extracted from the UniProtKB, HGNC and Ensembl databases [1–3] and were assigned to the human proteins from the reviewed part of UniProtKB. After extraction, both synonym expansion and restriction were performed. Multiple forms of expansion took place to increase the overall variation of synonyms. Expansion rules such as Greek letter normalisation, e.g. 'TNF α' would be expanded to 'TNF alpha', white-space substitution, 'TNF α' to 'TNF-α', and others took place depending on the synonym's features. Synonyms were eliminated based on multiple criteria. As an example, if the synonym was less than three characters long or the synonym could be found on a pre-compiled list of common English words, the synonym was discarded. After the expansion process, restriction was performed. If a synonym could not be found in PubMed it was discarded, thus resulting in a synonym set with more efficient entity recognition.

While automatic synonym extraction and expansion result in many meaningful synonyms, an extensive effort was put into curating them manually and checking the validity with special focus on common, short, shared, and interesting synonyms. New synonyms that were encountered during this process were added, if they were found to be specific. Contexts where certain synonyms were not valid were also identified and added as 'black lists', hereby limiting the number of false positives.

Similarly, synonyms for diseases were extracted from Disease Ontology [4] and MeSH [5], expanded, restricted, and curated.

A custom text mining engine implemented in C and Python was used for the actual text mining.

Fig. 1 shows the distribution of the number of abstracts mentioning each protein. It is clear that a small subset of proteins are very well studied, while most proteins are mentioned in only a few abstracts. This gives rise to a risk that text mining analyses be biased towards the few well-studied proteins.

## S1.2 Co-mentioning statistics

We computed co-mentioning statistics between, say, proteins and diseases by considering a protein $\pi$ and a disease $d$ and by counting the number of abstracts $n_\pi$ that mention the protein, the number of abstracts $n_d$ that mention the disease, and the number of abstracts $n_{\pi,d}$ that mention both of them. Together with the total number of abstracts $n$ we computed $p$-values for over-representation using Fisher's exact test [6] and the over-representation ratio

$$R(\pi, d) = \frac{n\, n_{\pi,d}}{n_\pi n_d}$$

which is the number of abstracts with synonyms for the two entities divided by the expected number if the entities were independently mentioned in $n_\pi$ and $n_d$ random

**Fig 1. Number of abstracts mentioning proteins.** The figure shows the number of abstracts for the 20 most described proteins. Only 174 proteins were mentioned in more than 10,000 abstracts, 1,595 proteins were mentioned in more than 1,000 abstracts, 6,630 proteins were mentioned in more than 100 abstracts, while the majority (13,780) were mentioned in more than 10 abstracts.

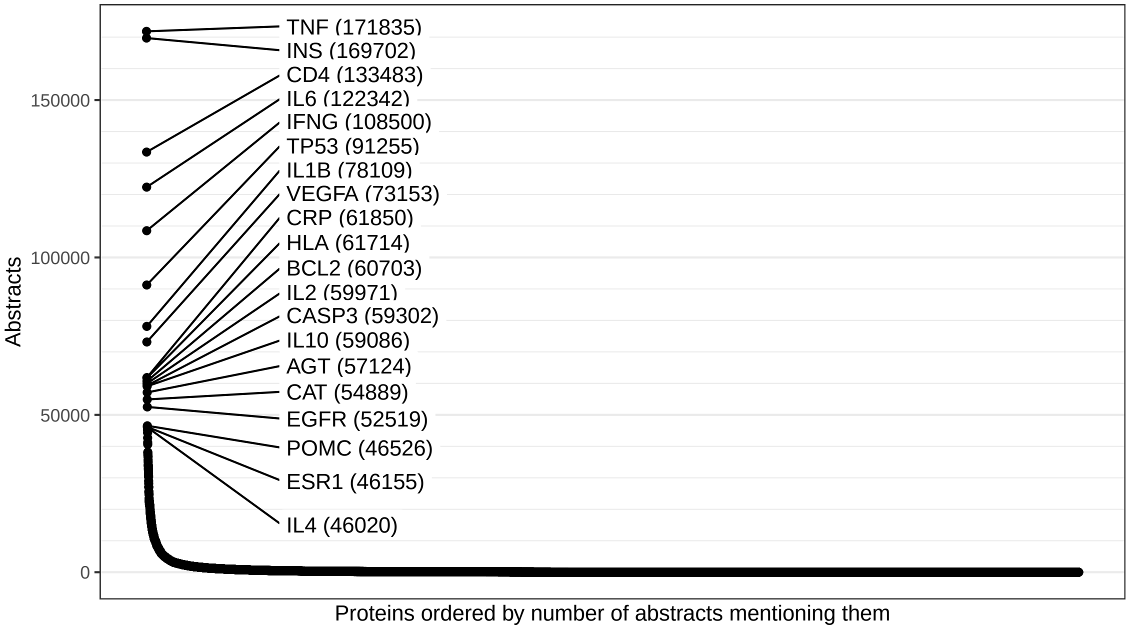


abstracts, respectively. An over-representation ratio above 1 indicates that the two entities are mentioned more often together than would be expected by random. As will be described in the following sections, co-mentioning statistics were used to identify the core T2D proteins and for clustering of $n$-grams into semantic concepts.

## S1.3 Identification of core T2D proteins

Text mining PubMed abstracts for T2D using over 100 synonyms, suggested and validated by our manual curation, finds more than 140,000 articles; many of which are related to the clinical challenges with treating T2D. As described in the previous section, we then applied a Fisher's exact test for over-representation for each protein, and we identified the 100 proteins with the lowest $p$-values. These 100 proteins, which we shall refer to as the '100 core T2D proteins' are provided in Table 1.

The 100 core T2D proteins were co-mentioned with T2D from 63 to 38,143 times, with JAZF1 being the least co-mentioned protein and insulin the most. A total of 47 of them were also part of either a) the KEGG pathway 'hsa04930' entitled 'Type II diabetes mellitus' [7–9], b) the similarly titled WikiPathways pathway 'WP1584' [10], or c) annotated in UniProt as involved in the disease 'Diabetes mellitus, non-insulin-dependent' (a synonym for T2D), indicating that the associations are well known, or even well established.

We reviewed the 100 core T2D proteins by manually inspecting recent abstracts

**Table 1.** Top 100 T2D associated proteins sorted alphabetically by gene name with UniProtKB accessions.

| Gene | UniProt | Gene | UniProt | Gene | UniProt | Gene | UniProt |
|---|---|---|---|---|---|---|---|
| ABCC8 | Q09428 | FABP4 | P15090 | IDE | P14735 | PPARGC1A | Q9UBK2 |
| ACE | P12821 | FFAR1 | O14842 | IGF1 | P05019 | PTPN1 | P18031 |
| ADIPOQ | Q15848 | FGF21 | Q9NSA1 | IGF2BP2 | Q9Y6M1 | PTPRN | Q16849 |
| ADIPOR1 | Q96A54 | FNDC5 | Q8NAU1 | IL6 | P05231 | PYY | P10082 |
| ADIPOR2 | Q86V24 | FOXO1 | Q12778 | INS | P01308 | RARRES2 | Q99969 |
| ADRB3 | P13945 | FTO | Q9C0B1 | INSR | P06213 | RBP4 | P02753 |
| AGER | Q15109 | G6PC | P35575 | IRS1 | P35568 | REN | P00797 |
| AGT | P01019 | GAD2 | Q05329 | IRS2 | Q9Y4H2 | RETN | Q9HD89 |
| AHSG | P02765 | GCG | P01275 | ITLN1 | Q8WWA0 | SELE | P16581 |
| AKR1B1 | P15121 | GCGR | P47871 | JAZF1 | Q86VZ6 | SERPINA12 | Q8IW75 |
| AKT1 | P31749 | GCK | P35557 | KCNJ11 | Q14654 | SERPINE1 | P05121 |
| ANGPTL8 | Q6UXH0 | GCKR | Q14397 | KCNQ1 | P51787 | SHBG | P04278 |
| APOA1 | P02647 | GHRL | Q9UBU3 | LEP | P41159 | SLC2A1 | P11166 |
| APOB | P04114 | GIP | P09681 | LEPR | P48357 | SLC2A2 | P11168 |
| APOC3 | P02656 | GIPR | P48546 | LPA | P08519 | SLC2A4 | P14672 |
| APOE | P02649 | GLP1R | P43220 | LPL | P06858 | SLC30A8 | Q8IWU4 |
| CAPN10 | Q9HC96 | GPR119 | Q8TDV5 | MGAM | O43451 | SLC5A1 | P13866 |
| CCL2 | P13500 | GSK3B | P49841 | MTNR1B | P49286 | SLC5A2 | P31639 |
| CD36 | P16671 | HBA1,HBA2 | P69905 | NAMPT | P43490 | SREBF1 | P36956 |
| CDKAL1 | Q5VV42 | HHEX | Q03014 | NOS3 | P29474 | TBC1D4 | O60343 |
| CETP | P11597 | HNF1A | P20823 | PDX1 | P52945 | TCF7L2 | Q9NQB0 |
| CRP | P02741 | HNF1B | P35680 | PON1 | P27169 | TNF | P01375 |
| CST3 | P01034 | HNF4A | P41235 | PPARA | Q07869 | UCP1 | P25874 |
| DPP4 | P27487 | HSD11B1 | P28845 | PPARD | Q03181 | UCP2 | P55851 |
| EEF1A2 | Q05639 | IAPP | P10997 | PPARG | P37231 | VCAM1 | P19320 |

co-mentioning synonyms for these proteins and synonyms for T2D. All the associations were deemed true, as we did not find any abstracts where a synonym for any of the 100 core proteins (or T2D) was used for another entity. This, we believe, is to a large degree due to our thorough automatic and manual curating of protein synonyms. In contrast, the Open Targets Platform [11] currently lists ladinin-1 as the second most associated T2D protein, likely due to confusion caused by its alternative name 'Linear IgA disease antigen', abbreviated 'LADA', which also abbreviates 'latent autoimmune diabetes mellitus in adults'.

## S1.4 Extracting $n$-grams from abstracts

We developed a pipeline to extract the $n$-grams from the raw PubMed abstracts. Typical non-alphanumeric characters (`#%-()[],.;:/=<>`) were stripped from the text before $n$-gram pattern detection to reduce trivial variation on $n$-grams. An example of a 3-gram is 'islet isolation method' consisting of 'islet', 'isolation', and 'method'. To reduce the number of generated $n$-grams and keep focus on useful patterns we removed $n$-grams consisting only of tokens resembling numbers and $n$-grams not found at least 10 times in PubMed.

We produced more than 10 million $n$-grams for $n = 1, \ldots, 7$ of different character lengths (see Fig. 2), and used the 1-grams to drive the generation of the $n$-grams for $n > 1$ as we identified common words such as 'is', 'in', 'of', 'with' etc. and discarded $n$-grams containing these words to avoid normal, but irrelevant, non-technical $n$-grams such as 'studies find evidence of'.

Finally we restricted the $n$-grams to those with at least 2 words as this resulted in a much more biological relevant set of $n$-grams, for example 'increased glucose' rather than simply 'glucose'. This produced a large number of naturally occurring, information rich $n$-grams with technical terms.

**Fig 2.** Statistics for all $n$-grams, $n \leq 7$. *Left:* Number of detected $n$-grams by $n$-gram character length. A 1-gram can be as short as one character, e.g. the number '1'. A 2-gram cannot be smaller than 3 characters since it is at least a character, a space and another character (e.g. 'p 0'). There are exponentially fewer $n$-grams for increasing $n$. *Right:* Scatterplot of the number of occurrences in PubMed per $n$-gram versus the $n$-gram's character length. The occurrence counts follow a power law distribution where $n$-grams for larger $n$ occur more rarely.

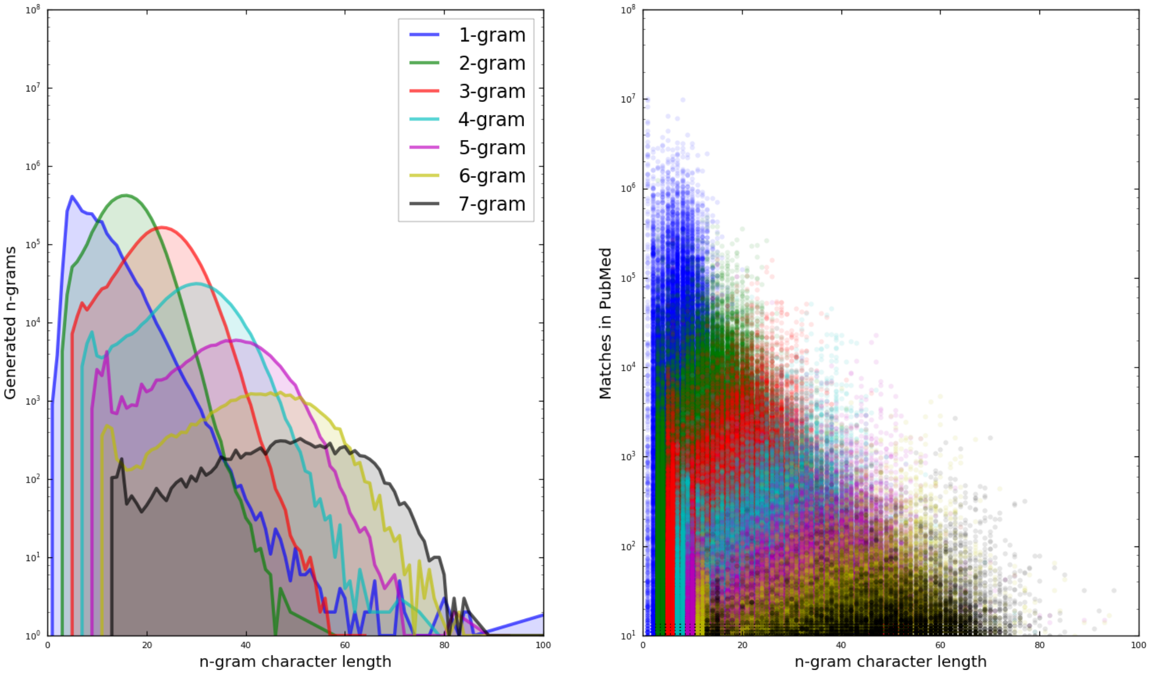


## S1.5 Homologous $n$-grams

We created a measure of text similarity between an $n$-gram $\bar{x}$ consisting of the tokens $x_1, \ldots, x_n$ and an $m$-gram $\bar{y}$ consisting of the tokens $y_1, \ldots, y_m$. We called this measure 'string homology' and we computed it by applying a customised algorithm outlined in the following. Inspired by BLOSUM matrices [12] we first constructed a similarity measure $h(x, y)$ between two individual tokens $x$ and $y$ based on a token length-normalised, inverse-character frequency score using the known characters frequencies in the English language. For each $i \in \{1, \ldots, n\}$ corresponding to a token $x_i$ we found the best matching token in $\bar{y}$, i.e. $y_j$ with $j = \arg\max_{j=1,\ldots,m}(h(x_i, y_j))$ and set $c_i = \frac{|X_i \cap Y_j|^2}{|X_i| \cdot |Y_j|}$ where $X_i$ and $Y_j$ were the sets of letters in the tokens $x_i$ and $y_j$,

respectively. We then computed

$$H(\bar{x}, \bar{y}) = \frac{1}{mn} \sum_{i=1}^{n} c_i^2$$

to produce the final, normalised string homology.

## S1.6 Semantic similarity of $n$-grams with same context

We constructed a similarity matrix where the rows and columns corresponded to an $n$-grams and an $m$-gram, respectively. The cells in the row for $n$-gram $\bar{x}$ and the column for $m$-gram $\bar{y}$ contained the over-representation ratio $R(\bar{x}, \bar{y})$ from text mining as described previously.

## S1.7 Clustering $n$-grams into semantic concepts

For each $n$-gram $\bar{x}$ and $m$-gram $\bar{y}$, $n, m = 2, \ldots, 7$ that were over-represented with T2D, we combined the above described string homology score $H(\bar{x}, \bar{y})$ and the over-representation ration $R(\bar{x}, \bar{y})$ into a single similarity matrix $M$ by an exponential weighting of each pair, $M(\bar{x}, \bar{y}) = H(\bar{x}, \bar{y})^a R(\bar{x}, \bar{y})^{(1-a)}$, where $a$ is a scaling factor that we set to $\frac{1}{2}$. Then, the MCL clustering algorithm [13] was applied to produce the semantic concepts as the clusters within this very large similarity matrix. We required two things in order for a pair of $n$-grams to be considered similar: First they must be similar in terms of having a similar wording (string homology) and secondly, they must be found in similar context.

Among the $n$-grams, 235,382 were over-represented with T2D and were clustered into 48,381 semantic concepts. Text mining for these resulted in 132 million occurrences with an average of 7.2 (and a median of 5) unique semantic concepts per abstract.

## S1.8 Article scoring

We constructed a co-mentioning statistics matrix with a row for each of the 100 core T2D proteins and a column for each of the semantic concepts. The cells of the matrix contained the positive pointwise mutual information, i.e. the logarithms (with base 10) of the over-representation ratios, so that a cell was 0, or close to 0, if there was no over-representation and larger than 0 if the protein and the semantic concept were mentioned together more often than expected by random. If a protein and a semantic concept were not mentioned together at all, or if they were mentioned together fewer times than expected by random, we put a 0 in the corresponding cell of the matrix. If $C$ denotes the set of semantic concepts and $\Pi$ the set of the 100 core T2D proteins, we constructed the matrix $W = (w_{\pi,c})_{\pi \in \Pi, c \in C}$, where $w_{\pi,c} = \max(0, \log_{10} R(\pi, c))$.

To score articles, we computed the average of all rows in the matrix, i.e. we set $w_c = \frac{1}{|\Pi|} \sum_{\pi \in \Pi} w_{\pi,c}$ for $c \in C$ and used the $(w_c)_{c \in C}$ vector as a set of weights that

each semantic concept contributed to an article's score, if the semantic concept was mentioned in the article. Thus, if $C_m$ denotes the set of semantic concepts mentioned in the title or abstract of the article with PMID $m$, we set

$$s(m) = \sum_{c \in C_m} w_c$$

to be the score of the article. Thus, an abstract would get a high score if semantic concepts often used together with the 100 core T2D proteins were mentioned. Note that mentioning the same semantic concept (or any of its $n$-grams) multiple times did not increase the score. Also, we did not normalise with the length of the abstract as abstracts are usually required to be of limited length, and thus longer abstracts could get a higher score by having more words.

The highest weight we found was 1.208 (see Table 1 in the main text) which was assigned to 'glucose homeostasis', with 21 semantic concepts having a weight of at least 1. Almost all the semantic concepts (47,297 out of 48,381) had a positive weight, however most of them were small (see Fig. 3), so it was not just a single semantic concept, or even a combination of concepts, that drove the article score, but rather the system looked for a large number of semantic concepts and then used their weights to determine article score.

**Fig 3. Histogram of weights for the semantic concepts used for article scoring.** A total of 47,297 semantic concepts out of 48,381 had positive weights.

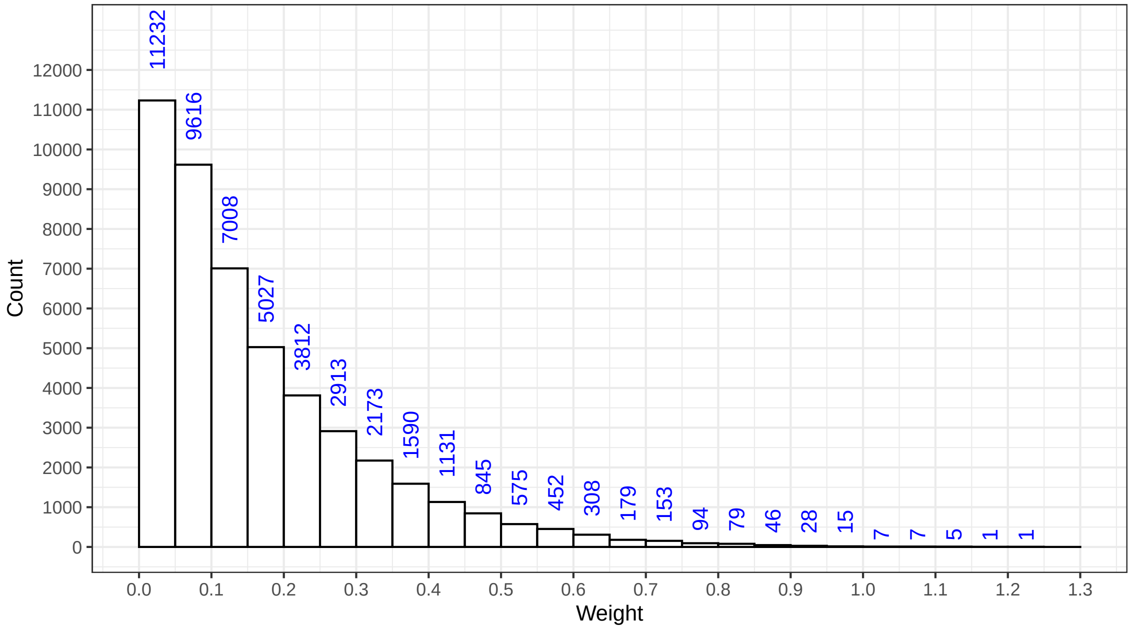


We found at least one semantic concept in most of the article abstracts from PubMed (see Fig. 4) with a concept containing the $n$-gram 'patients treated' being the most prevalent, but carrying a weight of zero. Although the number of concepts do correlate with the article score (data not shown), the histogram in Fig. 4) shows

that concepts are matched throughout PubMed abstracts across virtually all subjects, including T2D, where the highest weighted concepts are prevalent.

**Fig 4. Histogram of the number of semantic concepts detected in abstracts.** The average number of concepts is 7.3 per abstract (blue line), while the median is 5 concept per abstract. There were at least one concept detected for 88% of the 19.8 million included abstracts that had a title and a body.

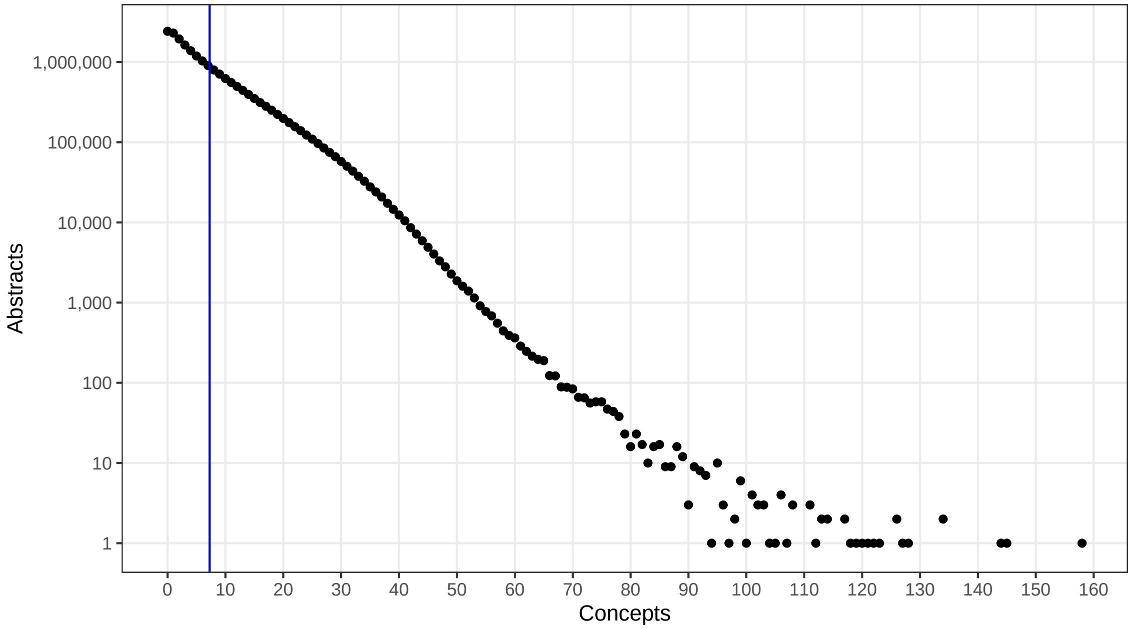


## S1.9 Scoring the T2D relevance of proteins

Each week, we found the highest scoring article, if any, for each protein $\pi$. Of these articles we then considered the 40 highest scoring published within the last 5 years. If $m_{1,\pi}, \ldots, m_{n_\pi,\pi}$ are the PMIDs of these articles ordered so that $s(m_{1,\pi}) \geq \cdots \geq s(m_{n_\pi,\pi})$, and where $n_\pi = 40$ unless the protein $\pi$ was mentioned in fewer articles, we computed the relevance score for the protein as

$$S(\pi) = \frac{1-\alpha}{\alpha} \sum_{k=1}^{n_\pi} \alpha^k s(m_{k,\pi})$$

where we used $\alpha = 0.9$. The motivation for this formula is that a) the highest scoring article contribute most, b) being mentioned in multiple high scoring articles increase the score, c) the contribution from additional articles is diminishing, and d) the contributions are spread out over time and not all come from, say, articles in a special issue of a journal (as they would all be published in the same week). The scaling factor $\frac{1-\alpha}{\alpha}$ stems from the fact that it is the inverse of the limit for $n \to \infty$ of $\sum_{k=1}^{n} \alpha^k$ for $-1 < \alpha < 1$, and since it scaled all protein scores equally it had no actual influence, but it ensured that a protein's score was always less than that of the highest scoring

article mentioning the protein. We imposed the limit $n_\pi \leq 40$ as $0.9^{40} \approx 0.0148$, and hence articles that were not among the 40 highest would contribute very little to the score.

## S1.10 High-jumping proteins

A rank of 1 was assigned to the highest scoring protein, and we considered a protein's change in rank to be positive, if it changed to a position nearer the top. The rankings by the scores stayed much the same from one week to the next due to the normalising effect of the sliding window computing the scores, as well as the fact that most proteins are not associated with T2D, and even if they are, they were rarely mentioned in a higher scoring article. Thus, in most cases the ranking of a protein only changed slightly, if at all, and sometimes only because some other protein(s) jumped higher, pushing the protein down a place, or two. Note that each weekly ranking went from 1 to 17,791, corresponding to the number of proteins we were able to find by text mining. Finally, note that ties were resolved in a deterministic but seemingly random way by ordering according to hash functions of the proteins' names.

To detect proteins that suddenly became strongly or comparatively stronger associated with T2D, we studied the empirical distribution of rank changes given the initial rank. This was used to assess the significance of the changes. Using a lead-in period to have the overall ranking of proteins reach a state where most had a stable rank, we recorded all positive and negative rank changes in the remaining period. More formally, we let $N$ be the number of proteins, let $\pi_i(r)$ denote the protein with rank $r$ in week $i$, and let $r_i(\pi)$ denote the rank in week $i$ of the protein $\pi$. We numbered the weeks after the lead-in period $1, \ldots, W$ and recorded for each possible rank $r \in \{1, \ldots, N\}$ the observed jumps $j_i(r) = r - r_{i+1}(\pi_i(r))$ for $i \in \{1, \ldots, W-1\}$, i.e. the change in rank for the protein with rank $r$ in week $i$ to the next week. We could then assess the significance of a jump $j$ places up from rank $r$ simply by counting the fraction of higher jumps we observed from rank $r$ previously, i.e.

$$p = \frac{1}{W-1} \left| \{ i \in \{1, \ldots, W-1\} \mid j_i(r) \geq j \} \right| .$$

To better distinguish significant high jumps we smoothed this distribution using a sliding window of size 51. A 'high-jumping protein' was one that recently jumped high enough with a low enough $p$-value according to this distribution.

# References

1. UniProt Consortium T. UniProt: the universal protein knowledgebase. Nucleic Acids Res. 2018;46(5):2699. doi:10.1093/nar/gky092.

2. Yates B, Braschi B, Gray KA, Seal RL, Tweedie S, Bruford EA. Genenames.org: the HGNC and VGNC resources in 2017. Nucleic Acids Res. 2017;45(D1):D619–D625. doi:10.1093/nar/gkw1033.

3. Zerbino DR, Achuthan P, Akanni W, Amode MR, Barrell D, Bhai J, et al. Ensembl 2018. Nucleic Acids Res. 2018;46(D1):D754–D761. doi:10.1093/nar/gkx1098.

4. Schriml LM, Mitraka E, Munro J, Tauber B, Schor M, Nickle L, et al. Human Disease Ontology 2018 update: classification, content and workflow expansion. Nucleic Acids Res. 2019;47(D1):D955–D962. doi:10.1093/nar/gky1032.

5. Medical Subject Headings; Available from: `https://www.nlm.nih.gov/mesh/`.

6. Fisher RA. On the Interpretation of $\chi^2$ from Contingency Tables, and the Calculation of $P$. Journal of the Royal Statistical Society. 1922;85(1):87–94. doi:10.2307/2340521.

7. Kanehisa M, Sato Y, Furumichi M, Morishima K, Tanabe M. New approach for understanding genome variations in KEGG. Nucleic Acids Res. 2019;47(D1):D590–D595. doi:10.1093/nar/gky962.

8. Kanehisa M, Furumichi M, Tanabe M, Sato Y, Morishima K. KEGG: new perspectives on genomes, pathways, diseases and drugs. Nucleic Acids Res. 2017;45(D1):D353–D361. doi:10.1093/nar/gkw1092.

9. Kanehisa M, Goto S. KEGG: kyoto encyclopedia of genes and genomes. Nucleic Acids Res. 2000;28(1):27–30.

10. Slenter DN, Kutmon M, Hanspers K, Riutta A, Windsor J, Nunes N, et al. WikiPathways: a multifaceted pathway database bridging metabolomics to other omics research. Nucleic Acids Res. 2018;46(D1):D661–D667. doi:10.1093/nar/gkx1064.

11. Koscielny G, An P, Carvalho-Silva D, Cham JA, Fumis L, Gasparyan R, et al. Open Targets: a platform for therapeutic target identification and validation. Nucleic Acids Res. 2017;45(D1):D985–D994. doi:10.1093/nar/gkw1055.

12. Henikoff S, Henikoff JG. Amino acid substitution matrices from protein blocks. Proc Natl Acad Sci USA. 1992;89(22):10915–10919.

13. Enright AJ, Van Dongen S, Ouzounis CA. An efficient algorithm for large-scale detection of protein families. Nucleic Acids Res. 2002;30(7):1575–1584. doi:10.1093/nar/30.7.1575.
